# Comparison of LPS-stimulated release of cytokines in punch versus transwell tissue culture systems of human gestational membranes

**DOI:** 10.1186/1477-7827-8-121

**Published:** 2010-10-15

**Authors:** Mark F Miller, Rita Loch-Caruso

**Affiliations:** 1 Department of Environmental Health Science, School of Public Health, University of Michigan, Ann Arbor, Michigan 48109, USA; 2 United States Environmental Protection Agency HQ, 1200 Pennsylvania Avenue NW, Mailcall: 2842T, Washington, DC 20460, USA

## Abstract

**Background:**

Cytokine signaling within the amnionic, chorionic and decidual extraplacental gestational membranes plays an important role in membrane rupture and the timing of birth. The predominant in vitro explant culture system for evaluating cytokine induction in human gestational membranes has been the free-floating biopsy punch culture. Punch systems have been used to investigate the impact of various toxicants, pharmaceuticals and genetic variation on expression of pro-inflammatory cytokines. More recently, a dual compartment transwell culture system has been developed that more closely mimics the intrauterine compartment. The current study compares these two systems with respect to release of pro- and anti-inflammatory cytokines in response to lipopolysaccharide (LPS), a model stimulant.

**Methods:**

Tissue samples were exposed to 100 ng/ml LPS for 12 h and cytokines were measured by ELISA. Data are expressed as increase relative to non-treated controls.

**Results:**

Levels of interleukin-6 increased in punch culture medium samples to a significantly greater extent (34.2 fold) compared with medium from transwell cultures in the amnion (6.6 fold) or choriodecidual (7.1 fold) compartments. Interleukin-8 also showed a significantly greater induction in punch (4.8 fold) than transwell amnion (1.6 fold) or choriodecidual (1.7 fold) samples. The anti-inflammatory interleukin-10 showed a significant difference between punch (36.5 fold) and transwell amnion (15.4 fold) samples, but no difference was observed between punch and transwell choriodecidual (28.5 fold) samples. Neither interleukin-1beta nor tumor necrosis factor-alpha (TNF-alpha) showed a significant difference between the punch and transwell samples.

**Conclusions:**

These results indicate that the pattern of LPS-stimulated cytokine release from gestational membranes in vitro depends on the culture system used, confounding comparisons of studies that use different gestational membrane culture systems to study inflammatory responses.

## Background

Gestational membranes (amnion, chorion laeve and decidua) collected immediately after birth and cultured in vitro allow assessment of responses in tissues with an intact cellular matrix. As such, cultures of human gestational membranes provide useful in vitro research models for inquiries into obstetric challenges such as inflammation, preterm premature rupture of membranes (PPROM) and preterm birth.

One model system used extensively to study stimulated production and release of cytokines in human gestational membranes in vitro involves explant culture of a biopsy punch, with the gestational tissue punch explant free-floating in culture medium. Biopsy punch explant cultures may use full-thickness membranes [1,2] as well as separated amnion [3,4] or choriodecidua [5,6]. This single-compartment explant culture system has been used to investigate cytokine [2-5,7-10], prostaglandin [4-6,8-11], adipokine [12] and protease [1,8] regulation.

In contrast to the free floating biopsy punch system, dual-compartment systems employ gestational membrane explants attached to rigid frames and suspended in culture medium, with discrete amniotic and choriodecidual chambers. Dual-compartment systems have used tissues mounted on a transwell insert [13-16], Ussing chamber [17], or other apparatus [18-20] to study cytokine and prostaglandin production induced by bacteria or bacterial toxins [13,14,16,17,21,22], yeast [15] or oxygen tension [18], as well as meconium interactions with the gestational membrane [19]. The two-compartment system has provided a model system in which investigators can expose and sample medium from each side of the membranes independently.

To date no reports have directly compared the cytokine responses of traditional free-floating biopsy punch explants and transwell-mounted explant cultures. The potential for different responses due to model-related restrictions suggests a need for further investigation. Therefore, the aim of this study is to compare floating biopsy punch and transwell mounted explant culture systems for lipopolysaccharide (LPS)-induced cytokine release.

## Methods

### Sample collection

Human extraplacental gestational membranes were obtained from healthy non-laboring women undergoing scheduled caesarean section deliveries at 37-39 completed weeks gestation at the University of Michigan Women's Hospital Birth Center in Ann Arbor, Michigan. Exclusion criteria included cigarette smoking, prescription of antibiotics in the past two weeks, collagen vascular disease, immunocompromised conditions, bacterial vaginosis or clinical chorioamnionitis (as noted in the chart or suspected by attending physician), cervical cerclage, third trimester bleeding, major maternal medical conditions (e.g., chronic renal disease, sarcoidosis, hepatitis, HIV), pre-eclampsia, diabetes, multifetal pregnancy, or any other condition which would require the tissue to undergo pathological evaluation. The investigators had no direct interaction with the human subjects and the tissues collected would have been otherwise discarded. Personal identifiable information was not collected, in compliance with the University of Michigan Institutional Review Board requirements.

Immediately after delivery, gestational membranes were dissected from the placental disk and transported to the laboratory in warm isotonic Dulbecco's phosphate buffered saline (DPBS; Invitrogen, Grand Island, NY). Full thickness membranes were dissected away from the placental disk, maintaining a minimum 3-cm margin to prevent sampling from the transitional zone. A 3-cm margin was also maintained around the site of incision and any areas where the membranes may have been damaged or separated. Laboratory practices followed universal safety precautions for handling human tissue (e.g., personnel vaccination for hepatitis B and wearing of laboratory safety glasses, gloves, face mask and lab coat when handling tissues).

### Culture and treatment

The various culture studies cited in this report employ a wide variety of methods. Variations among LPS source and concentration, media volume, tissue mass, and duration of exposure can have great impacts on the measured cytokine release. The methods used in this study were established to minimize the effect of these variables between culture systems. Medium volumes in the transwell culture system were chosen based on previous reports and were designed to keep the fluid level even on both sides of the suspended membrane. By keeping the fluid levels even we eliminated hydrostatic pressure between chambers. The volume of medium in the punch culture system was then set to approximate the transwell culture system, and cytokine concentrations were adjusted based on tissue weight.

Full-thickness gestational membrane explants were established using punch or transwell-mounted culture methods. Under sterile conditions, intact membranes were rinsed with isotonic PBS to remove blood, and randomly sampled for 12-mm punches and 3-cm × 3-cm squares of tissue for use in transwell-mounted cultures. From each of seven gestational membranes, twelve punch explants and eight transwell explants were collected from throughout the membranes.

Culture medium consisted of Dulbecco's Modified Eagle Medium (DMEM) (Gibco, Grand Island, NY) supplemented with 100 units/ml penicillin (HyClone, Logan, UT), 100 ug/ml streptomycin (HyClone, Logan, UT) and 1% heat-inactivated fetal bovine serum (FBS) (Gibco, Grand Island, NY). Individual punches were free-floated in 12-well polystyrene tissue culture plates containing 1 ml of medium (Becton Dickinson, Franklin Lakes, NJ). Transwell cultures were established using previously published methods [16]. For each transwell culture, a 3-cm × 3-cm square of tissue was mounted on a polycarbonate transwell insert (gift from Corning Inc., Corning, NY) using an elastic latex band, with the choriodecidua facing the inner chamber of the transwell insert and the amnion facing the outer chamber. Excess tissue extending beyond the elastic band was trimmed and each transwell insert was suspended in a culture well, creating two discrete chambers. Culture medium was added to each transwell chamber, with an inner chamber medium volume of 0.5 ml and an outer chamber medium volume of 1.5 ml, providing equal depth of the medium across the two chambers.

Cultures were acclimated for 24 h, with medium changes after 6 h and 22 h. After acclimation, six punch and four transwell cultures from each subject were randomly assigned to control or lipopolysaccharide (LPS) treatment groups. Just prior to the experiment, an LPS stock solution (100 μg/ml LPS in sterile, deionized water) was diluted with culture medium to a final exposure concentration of 100 ng/ml LPS (from *Salmonella typhimurium*; List Biological Laboratories, Inc., Campbell, CA). The concentration of 100 ng/ml LPS was used because in prior experiments this concentration elicited maximal release of TNF-α and IL-8 in transwell cultures of human gestational membranes [16]. To initiate the experiment, the medium was exchanged with either fresh culture medium (controls) or medium containing LPS. In transwell cultures, LPS exposure medium was added to both the amniotic-facing and the choriodecidual-facing chambers. Based on preliminary studies, 12 h was selected as the exposure duration to provide a measurable response in all cytokines tested. Control cultures were exposed to culture medium without LPS. All cultures were maintained in a 95% air/5% CO_2 _atmosphere at 37 ± 0.5°C.

### Cytokine assessment

Concentrations of the cytokines IL-1β, IL-6, IL-8, IL-10 and TNF-α were quantified by ELISA using the manufacturer's suggested protocol (Duosets, R&D systems, Minneapolis, MN) in the Immunology Core Facility at the University of Michigan Cancer Center. Immunohistochemistry (IHC) was performed at the University of Michigan Research Histology & Immunohistochemistry Laboratory. Samples for IHC analysis were fixed in 10% formalin and stored in 70% ethanol until processing. Samples were embedded in paraffin, sectioned and labelled with anti-IL-8 polyclonal primary antibody (Abcam Inc., Cambridge, MA) and biotinylated secondary antibody (Abcam Inc., Cambridge, MA). Negative controls were performed to assure antibody specificity in this tissue.

### Data analysis

The cytokine ELISA data are represented as concentration mean ± SEM for each treatment within a culture method. Because the culture methods used different tissue weights and medium volumes, data are also represented as fold-increase over control to allow comparison of LPS activation between culture methods (mean ± SEM). Differences between control and LPS-stimulated groups were analyzed using t-tests or Mann-Whitney rank sum tests for data sets that were not normally distributed. Differences between culture methods for fold increases of response were analyzed using one-way analysis of variance (ANOVA) with Student-Newman-Keuls method for posthoc pairwise comparisons of means. A *p*-value < 0.05 was considered statistically significant.

## Results

Table 1 provides the cytokine concentrations in the medium of control and LPS-exposed human gestational membranes in biopsy punch and transwell cultures. Non-treated controls showed no differences in basal release of IL-1β, IL-6, IL-10 and TNF-α between the amniotic and choriodecidual compartments of the transwell cultures. In contrast, IL-8 had greater unstimulated secretion into the choriodecidual compartment (236.48 ± 32.46 ng/ml) than into the amniotic compartment (154.30 ± 29.15 ng/ml) in untreated controls (p < 0.001). LPS induced a significant increase of IL-1β, IL-6, IL-8, IL-10, and TNF-α in the tissue punch cultures as well as on each side of the transwell cultures compared with respective controls (p < 0.05; Table 1).

**Table 1:** In vitro cytokine secretion into culture medium by human gestational membranes using a biopsy punch explant culture system or a transwell mounted explant culture system^a^

	Punch		Transwell Amnion		Transwell Choriodecidua	
	Control	LPS^b^	Control	LPS^b^	Control	LPS^b^
**Interleukin-1β**	10.66	66.50	30.54	203.10	36.72	126.72
(pg/ml)	(1.68)	(13.87)	(10.40)	(81.60)	(18.91)	(42.35)
**Interleukin-6**	4.42	78.83	15.22	80.34	23.75	104.19
(ng/ml)	(1.24)	(20.27)	(5.33)	(30.10)	(7.33)	(23.81)
**Interleukin-8**	62.41	225.37	154.30^c^	238.46	236.48^c^	377.94
(ng/ml)	(14.23)	(48.57)	(29.15)	(43.81)	(32.46)	(55.59)
**Interleukin-10**	12.61	335.11	15.42	166.57	15.34	290.66
(pg/ml)	(2.78)	(50.05)	(4.21)	(69.59)	(4.13)	(70.21)
**TNF-α**	21.37	5026.48	33.11	8394.66	50.04	11109.32
(pg/ml)	(3.44)	(1419.42)	(4.31)	(4192.91)	(13.50)	(4789.19)

Abbreviations: LPS, lipopolysaccharide; TNF-α, tumor necrosis factor-α

^a ^Cytokines were measured 12 h after incubation with or without 100 ng/ml LPS. In transwell cultures, LPS was added to both sides of the membranes and the cytokine concentrations are provided separately for the amniotic and choriodecidual compartments. Data are presented as mean (SEM). (N= 7 membranes)

^b ^LPS treatments induced a significant increase of all cytokines compared with their respective controls (P < 0.05)

^c ^Basal secretion of interleukin-8 differed significantly between the amniotic and choriodecidual sides of the transwell culture (P < 0.001)

**Figure 1:**
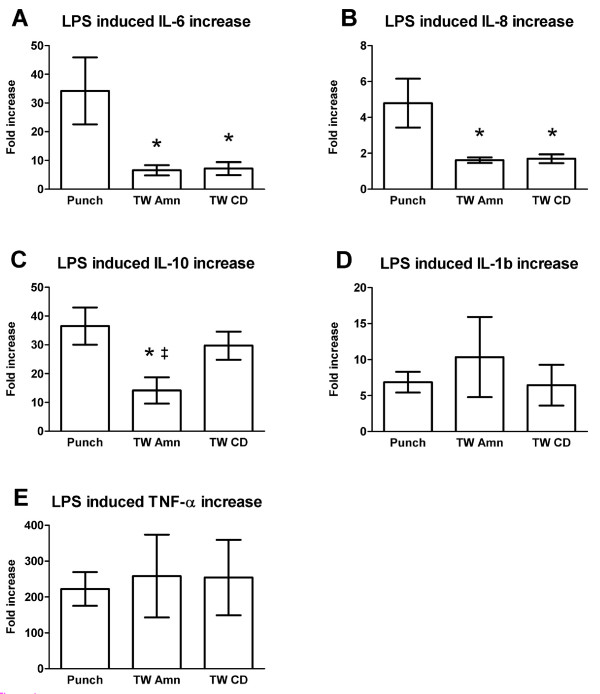
**Lipopolysaccharide (LPS)-induced release of cytokines from human gestational membranes in vitro**. Lipopolysaccharide (LPS)-induced release of (A) interleukin-6 (IL-6), (B) interleukin-8 (IL-8), (C) interleukin-10 (IL-10), (D) interleukin-1β (IL-1β), and (E) tumor necrosis factor-α (TNF-α) into culture medium of punch and transwell cultures. For the transwell cultures, the cytokine responses are shown for the amniotic chamber (TW Amn) and the choriodecidual chamber (TW CD). Data are represented as fold increase over non-treated control samples ± SEM. Transwell culture cytokine increases are provided separately for the amniotic and choriodecidual compartments. *Indicates significant difference compared with punch cultures (P < 0.05). ‡Indicates significant difference compared with TW-CD (P < 0.05). N = 7 membranes.

By representing LPS induction within a treatment model as a fold increase over non-treated control, we allow a comparison between treatment models that is not biased by variations in tissue mass or medium volume (Figure 1). On the basis of relative increase over control, LPS induced secretion of both IL-6 (Figure 1A) and IL-8 (Figure 1B) to a greater extent in the punch culture system (34 and 4.8 fold, respectively) than in either compartment of the transwell culture system (amniotic compartment, 6.6 and 1.6 fold increase for IL-6 and IL-8, respectively; choriodecidual compartment, 7.1 and 1.7 fold increase for IL-6 and IL-8, respectively) (p < 0.05). Likewise, a greater LPS-stimulated IL-10 response was observed in the punch cultures (36-fold increase) compared with the amniotic side of the transwell culture (14-fold increase); (p < 0.05); however, the response on the choriodecidual side of the membranes (30-fold increase) was similar to that of the punch cultures and significantly greater than that of the amniotic side of the membranes (p < 0.05) (Figure 1C). There were no statistically significant differences in the relative response to LPS stimulation for IL-1β (Figure 1D) and TNF-α (Figure 1E), comparing secretion into the medium by membranes in transwell cultures and punch cultures.

**Figure 2:**
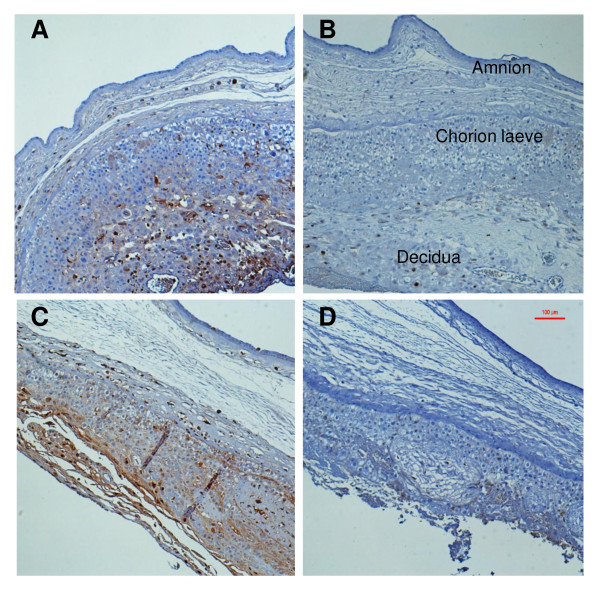
**Immunohistochemical detection of interleukin-8 (IL-8) in human gestational membranes**. Free-floating punch explants cultures (A and B) or transwell-mounted explants cultures (C and D) in the presense of lipopolysacchride (LPS) (A and C) or absence of LPS (B and D). Increased expression of IL-8 was identified within regions of the chorion laeve, decidua and, to a lesser extent, the amniotic mesenchyme, of LPS-exposed free-floating punch explants (A) and transwell-mounted explants (C) compared with respective non-treated controls (B and D).

Immunohistochemical labeling of IL-8 was performed on paraffin-embedded sections to identify cellular origins of this cytokine within the amnion, chorion laeve and decidua. IL-8 was selected for evaluation due to its significant secretion difference between punch cultures and both compartments of the transwell cultures. Figure 2 shows representative immunohistological sections of control and LPS-treated membranes from punch and transwell culture systems. Unstimulated punch and transwell membranes showed limited IL-8 positive labeling localized intracellularly within the cytoplasm of histiocytes. After stimulation with LPS, marked increases of intracellular IL-8 were visualized in the chorion leave and decidua; less marked increases were observed in the amniotic mesoderm.

## Discussion

Cytokines are important mediators of cell signalling within the human gestational membranes. Pro-inflammatory cytokine genes are up-regulated during labor [23,24], leading to increased cytokine secretion and triggering the induction of prostaglandins [25] and matrix metalloproteinases [26] that stimulate uterine contraction [27], cervical dilation [28] and the rupture of the gestational membranes [29]. Elevated IL-1β, IL-6 and TNF-α levels in the amniotic fluid are associated with both term [30] and preterm [25] parturition.

In vitro cytokine secretion into explant culture medium has been widely used as a measure of inflammation for human gestational membranes [2,4,5,7,8,11,13,22]. Two popular model systems for the study of human gestational membranes in vitro are the biopsy punch explant culture system and the transwell-mounted explant culture system. In the present study, we compared LPS-stimulated cytokine release for these two model systems.

Regardless of culture method, each of the cytokines assessed in the present study (IL-1β, IL-6, IL-8, IL-10, and TNF-α) increased in response to LPS compared to respective controls. However, when expressed as fold-increase, different patterns of LPS-stimulated response became apparent. Specifically, stimulation with LPS increased secretion of IL-6 and IL-8 to a greater extent in punch explant cultures compared with either the amniotic or choriodecidual responses assessed in transwell cultures. Differences between the two culture systems were observed for the anti-inflammatory IL-10, also, with the increase of IL-10 concentrations greater in the punch explant cultures compared with the amniotic but not the choriodecidual responses in transwell cultures. Although both systems have been widely used, the present report is the first direct comparison of the culture systems and demonstrates that the pattern of LPS-stimulated cytokine secretion differs between these culture methods.

Consistent with previously published reports using two-compartment tissue culture systems [2,7,13,16,17], elevated secretion was observed in response to endotoxic LPS stimulation for all cytokines tested (IL-1β, IL-6, IL-8, IL-10 and TNF-α). Elevated IL-6 concentrations in the amniotic fluid have been used as a marker of gestational membrane inflammation [31] and are associated with preterm labor [32] and PPROM [33]. After stimulation with LPS, the medium concentrations of IL-6 were five-fold greater in punch cultures when compared to transwell secretion into the amniotic or choriodecidual chambers. The IL-6 medium concentrations suggest an amplified cytokine response to LPS in the punch cultures, possibly due to positive feedback regulation of IL-6.

Previous reports have identified the choriodecidua, and specifically the decidual and chorionic trophoblasts [34,35], as the primary site of IL-6 production in non-laboring human gestational membranes, with little or no production in the amnion [4,10]. In the latter studies, LPS (5 ug/ml) increased IL-6 secretion three fold from separated choriodecidual explants whereas there was no effect in separated amniotic explants.

In the present study, we observed similar levels of IL-6 in the amniotic and choriodecidual media, as did Zaga-Clavellina et al. after 24 h of stimulation with cervicovaginal-derived *E. coli *[14]. In a previous report from our laboratory, concentrations of IL-6 were higher in choriodecidual chamber medium than amniotic chamber medium of transwell cultures after 8 h of stimulation with 100 ng/ml LPS; [16] it is possible that differences in duration of exposure may explain this difference in IL-6 response. Keelan et al. observed greater LPS-induced IL-6 and TNF-α increases in the choriodecidual medium when compared to the amniotic medium after an 8 h exposure in Ussing chambers; however, no significant IL-8 stimulation was observed [17]. It should be noted that the latter Ussing chamber experiment used different time-points and continuous perfusion to a high concentration of LPS (5 ug/ml) on only the choriodecidual face of the membrane, which may have contributed to the differences in IL-8 and TNF-α compared to the present study. Cytokines have been previously shown to cross the gestational membranes in vitro but at rates much lower than those necessary to allow equal choriodecidual and amniotic levels of IL-6 after 12 h of stimulation [20]. Therefore, we suggest that IL-6 crosses the choriodecidual-amniotic barrier at rates much greater than previously suggested, at least in the transwell culture system used in our study.

The LPS-induced 4.8-fold IL-8 increase we observed in the punch explant cultures is similar to the 3.4-fold increase reported by Menon et al. after 24-h stimulation with 100 ng/ml LPS using a punch explants from intact membranes [36]. Moreover, the mean IL-8 concentration seen in the present study was nearly three-fold greater in punch cultures compared with transwell culture medium in the amniotic or choriodecidual chambers. Concentrations of IL-8 increased to a similar extent in the amniotic and choriodecidual chambers of the transwell cultures of the present experiment, consistent with immunohistochemical evidence of LPS stimulation of IL-8 production in all three layers of the gestational membranes. However, in contrast to the similar amniotic and choriodecidual responses observed in the present study, LPS increased IL-8 concentrations on the choriodecidual side only of transwell-mounted gestation membranes exposed to LPS for 8 h in a previous study from our laboratory [16]. Differences between the two studies may be related to different durations of exposure to LPS; additionally, staining for IL-8 was more intense and dense in chorionic and decidual tissues compared with the amnion, suggesting that differences in thickness of the choriodecidual layers of tissues may have contributed to the different responses observed between the two experiments. It should be noted that the modest (approximately 1.6- fold) increases represent relatively large changes in release and local concentration due to the high unstimulated secretion of IL-8. Furthermore, increased IL-8 concentrations were observed in the choriodecidual compartment medium compared to the amniotic compartment medium in unstimulated control transwell cultures, suggesting directional secretion of cytokines from the human gestational membranes. The qualitative histological findings in this study support the cytokine measurement data, with positive IL-8 labeling observed primarily in the decidual region of the untreated transwell-cultured tissues. The ability of IL-8 to cross the gestational membranes as a functional protein has yet to be investigated.

The LPS-induced secretion of IL-10 was greater on the choriodecidual side compared to the amniotic side of membranes in transwell cultures, consistent with a previous report [16]. IL-10 is an important anti-inflammatory cytokine, inhibiting immune function and suppressing pro-inflammatory cytokine production [37]. Induced by other cytokines and prostaglandins [37,38], IL-10 has emerged as a possible mediator of pregnancy maintenance [39]. Recent reports show that IL-10 null mutant mice are more susceptible to LPS-induced fetal loss (a model for preterm delivery) than wild-type mice, and administration of exogenous IL-10 protects both IL-10 knockout mice and wild-type mice from fetal loss [40]. Moreover, IL-10 null mutant mice are more susceptible to toxicant (polychlorinated biphenyl)-induced preterm birth (birth on gestation day 17) compared with wild-type mice [41]. Preferential secretion of IL-10 from the choriodecidual face of the gestational membranes, as seen in this study, suggests that gestational membranes may play an important role in maintaining pregnancy.

We suggest that the differences in cytokine response observed in transwell cultures versus punch explant cultures reflect the lack of compartmentalization in the punch explant system. In contrast to the single compartment punch system, the transwell culture system maintains separated amniotic and choriodecidual compartments that more closely mimics the physiologic separation of fetal and maternal compartments in vivo. Because cytokines are regulated through receptor-mediated activation of second messenger and nuclear transcription pathways that are susceptible to positive feedback [42], secretion of cytokines into the single-compartment punch system may allow direct signalling between the amnion and choriodecidua through the shared medium, leading to increased positive feedback and causing the cytokine surge seen at 12 h. In contrast, the two-compartment transwell culture system prevents signalling through the medium between the amnion and the choriodecidua, thereby maintaining the physiologic condition of separated fetal and maternal compartments. Regardless, the results of the present study show that different gestational membrane culture systems can yield different magnitudes of response to an inflammatory stimulus, such that caution should be used in making comparisons of studies that use different culture systems.

## Competing interests

The authors declare that they have no competing interests.

## Authors' contributions

Both MFM and RL-C have made substantial contributions to conception and design, acquisition of data, and analysis of data. Both have been involved in drafting the manuscript or revising it critically. All authors read and approved the final manuscript.
